# New Insights into Orphan Nuclear Receptor SHP in Liver Cancer

**DOI:** 10.11131/2015/101162

**Published:** 2015-08-18

**Authors:** An Zou, Sarah Lehn, Nancy Magee, Yuxia Zhang

**Affiliations:** Department of Pharmacology, Toxicology & Therapeutics, University of Kansas Medical Center, Kansas City, KS 66160, USA

**Keywords:** Nuclear receptors, small heterodimer partner, metabolic disease, liver cancer

## Abstract

Small heterodimer partner (SHP; NR0B2) is a unique orphan nuclear receptor (NR) that contains a putative ligand-binding domain but lacks a DNA-binding domain. SHP is a transcriptional corepressor affecting diverse metabolic processes including bile acid synthesis, cholesterol and lipid metabolism, glucose and energy homeostasis, and reproductive biology via interaction with multiple NRs and transcriptional factors (TFs). Hepatocellular carcinoma (HCC) is one of the most deadly human cancers worldwide with few therapeutic options and poor prognosis. Recently, it is becoming clear that SHP plays an antitumor role in the development of liver cancer. In this review, we summarize the most recent findings regarding the new SHP interaction partners, new structural insights into SHP’s gene repressing activity, and SHP protein posttranslational modifications by bile acids. We also discuss the pleiotropic role of SHP in regulating cell proliferation, apoptosis, DNA methylation, and inflammation that are related to antitumor role of SHP in HCC. Improving our understanding of SHP’s antitumor role in the development of liver cancer will provide new insights into developing novel treatments or prevention strategies. Future research will focus on developing more efficacious and specific synthetic SHP ligands for pharmaceutical applications in liver cancer and several metabolic diseases such as hypercholesterolemia, obesity, diabetes, and fatty liver disease.

## 1. Introduction

Nuclear receptors (NRs) constitute a large family of transcription factors (TFs) that govern a wide array of cellular activities in development, metabolism, and physiology. The NR genes are commonly divided into three classes: the endocrine, adopted orphan, and orphan receptors according to their physiological ligands and potential function. Dysfunction of NR signaling leads to a wide spectrum of metabolic diseases including obesity, diabetes, fatty liver disease, and cancers [1].

**Figure 1:**
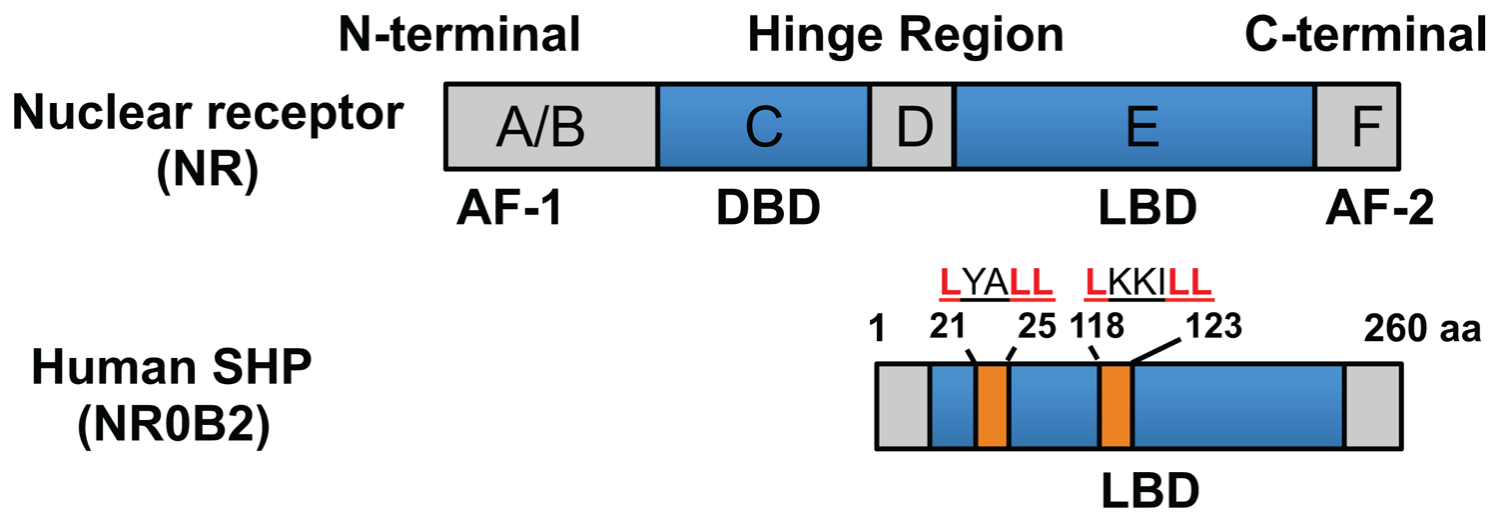
Domain structures of a typical nuclear receptor (NR) and human SHP. Top: the classical NR contains an N-terminal ligand-independent transactivation domain (A/B domain), a DNA binding domain (DBD or C domain), a hinge region (D domain), a ligand-binding domain (LBD or E domain), and a C-terminal ligand-dependent transactivation domain (AF2 or F domain). Bottom: SHP contains two dimerization motifs (residues 21–25 and 118–123) and a LBD but lacks a DBD. SHP represses gene transcription through interaction with the other NRs or TFs by utilizing two functional LXXLL-related motifs that are located in the putative N-terminal helix 1 and C-terminal helix 5.

Classical NRs contain five functional domains: an N-terminal ligand-independent transactivation domain, DNA-binding domain (DBD), hinge region, ligand-binding domain (LBD), and a C-terminal ligand-dependent transactivation domain (Figure 1). The presence of a putative LBD classifies small heterodimer partner (SHP; NR0B2 for nuclear receptor subfamily 0, group B, member 2) as a member of the NR family, although its endogenous ligand is currently unknown. SHP lacks a DBD but represses gene transcription by acting as a corepressor. SHP inhibits numerous NRs and TFs in diverse metabolic pathways including bile acid synthesis, cholesterol and lipid metabolism, glucose metabolism, and energy homeostasis [2–8].

Hepatocellular carcinoma (HCC) is the most rapidly increasing cause of cancer death in the United States [9]. HCC development is linked to the metabolic disease as both increased cancer risk and worsened cancer outcome have been observed in metabolic syndrome and nonalcoholic fatty liver disease [10]. Recent studies showed that SHP plays an antitumor role in the development of liver cancer. Extensive reviews regarding the role of SHP in regulating metabolism and metabolic diseases have been addressed elsewhere [11, 12]. In this review, we summarize the most recent findings regarding the new SHP interaction partners, new structural insights into how SHP represses gene transcription, and SHP protein posttranslational modifications (PTMs) mediated by bile acids. We also discuss the pleiotropic role of SHP in regulating cell proliferation, apoptosis, DNA methylation, and inflammation that are related to antitumor role of SHP in the development of liver cancer. An increased understanding of tumor suppressor function of SHP in the development of liver cancer will provide critical knowledge to improve the diagnostic, therapeutic, or preventive strategies for liver cancer.

## 2. Progress in the Identification of SHP Interaction Partners

The SHP gene is located on human chromosome 1p36.1 and mouse chromosome 4. It is composed of two exons interrupted by a single intron spanning approximately 1.8 kilobases (kb) in humans and 1.2 kb in mice [13]. Because SHP lacks a DBD, instead of binding directly to its target genes, SHP heterodimerizes with many DNA-bound NRs through its two functional LXXLL-related motifs (also called NR-boxes) located in the putative N-terminal helix 1 and C-terminal helix 5 (Figure 1) [14].

Through interaction with a board array of NRs and TFs, SHP can influence multiple target genes involved in diverse biological processes. To date, about half of mammalian NRs and several TFs interact with SHP. This includes liver receptor homologue-1 (LRH-1), hepatocyte nuclear receptor 4*α* (HNF4*α*), estrogen receptors (ERs), estrogen receptor-related receptors (ERRs), liver X receptors (LXRs), peroxisome proliferator-activated receptors (PPARs), glucocorticoid receptor (GR), thyroid hormone receptor *β* (TR*β*), retinoic acid receptor *α* (RAR*α*), farnesoid X receptor (FXR), pregnane X receptor (PXR), constitutive androstane receptor (CAR), androgen receptor (AR), nerve growth factor IB (NGFI-B or Nur77), and common heterodimerization partner retinoid X receptors (RXRs) [11, 12]. Recently, several new interaction partners for SHP have been identified. One study identifies a previously unrecognized role of neuronal PAS domain-containing protein 2 (Npas2) and SHP crosstalk in maintaining the oscillation of liver lipid metabolism. In this study, SHP inhibits Npas2 gene transcription through interaction with retinoic acid-related orphan receptor (Ror) [15]. Another study shows that SHP interacts with forkhead box A1 (FOXA1) in the regulation of betaine-homocysteine S-methyltransferase (Bhmt) transcription, thus playing a role in maintaining oscillatory homocysteine homeostasis [16]. SHP also interacts with lysine-specific histone demethylase 1 (LSD1) which is induced by FXR, and the recruitment of LSD1 to BA synthetic genes Cyp7a1 and Cyp8b1 and BA uptake transporter gene Ntcp is important for gene repression [17]. This study identifies LSD1 as a novel histone-modifying enzyme in the reduction of hepatic BA level mediated by the FXR and SHP.

## 3. New Structural Insights into the Transcriptional Repression by SHP

**Figure 2:**
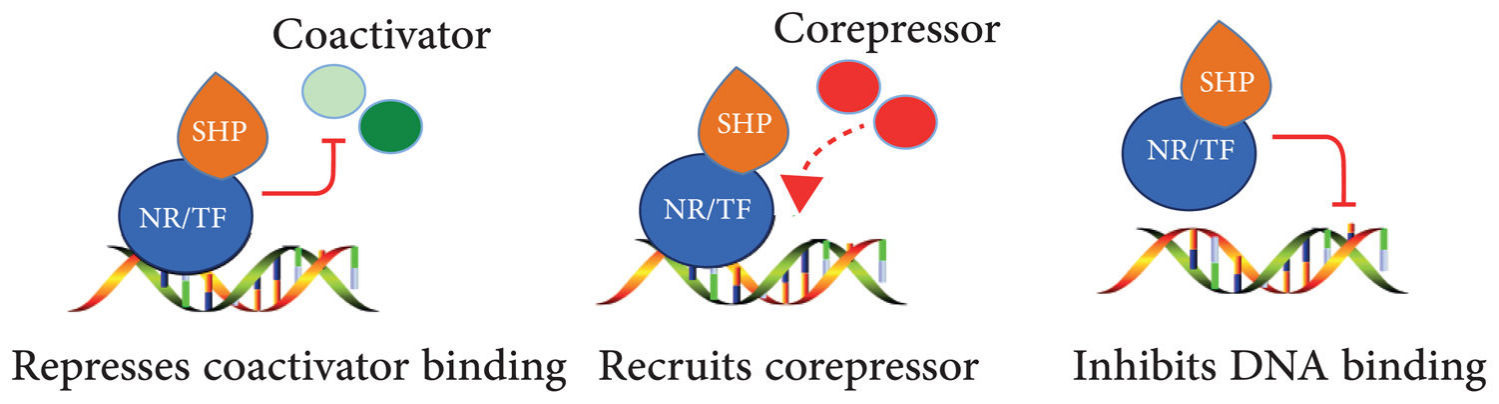
SHP inhibits gene transcription. SHP represses nuclear receptor (NR) or transcription factor (TF)-mediated transactivation by competing for coactivator binding, the recruitment of corepressors, and directly inhibiting NR or TF binding DNA. Activation is shown as a green arrow and inhibition is shown as a red line.

The molecular basis for ligand-dependent NRs regulated gene transcription is well known. In detail, ligand-activated NR recruits coactivators that contain LXXLL motifs to its C-terminal activation function-2 (AF-2) helix (also called helix H12) located in the LBD [18]. In contrast, antagonist destabilizes the AF-2 helix from the canonical LBD fold, thus opening up an extended groove for interactions with the LXXXLXXX motifs in NR corepressors [19]. As a unique transcriptional repressor without a known endogenous ligand, the precise mechanism whereby SHP represses transcription remains unclear; however, from recent studies, three mechanisms have been proposed (Figure 2). In the first proposed mechanism, SHP binds to the AF-2 helix of NRs through two LXXLL motifs located in the N-terminal helix 1 and C-terminal helix 5 [11, 14], resulting in the direct competition with coactivator binding to NR. In the second proposed mechanism, SHP acts as a direct transcriptional repressor by recruiting conventional corepressors such as E1A-like inhibitor of differentiation (EID1), mSin3A/HDAC corepressors, class III histone deacetylase SIRT1, G9a methyltransferase, and Brm-containing Swi/Snf remodeling complex, resulting in sequential modifications at the target gene promoter which inhibit gene transcription [20–23]. Lastly, the third mechanism proposes that SHP binds to NRs resulting in the dissociation of the SHP-NRs complex from certain promoters. Some examples in this mechanism include SHP repression of RAR-RXR heterodimers, RAR-PXR heterodimers, agonist-dependent ER*α* dimerization, and HNF4*α* homodimerization [8, 24–26]. Additionally, SHP interacts with some TFs including agonist-activated arylhydrocarbon receptor (AHR)/AHR nuclear translocator (ARNT), HNF-3, JunD, and CCAAT/enhancer-binding protein *α* (C/EBP*α*) and inhibits their DNA binding and transactivation properties [27–30].

Although most studies reveal SHP as a transcriptional repressor, SHP also activates nuclear factor-*κ*B (NF-*κ*B) in oxidized low-density lipoprotein (oxLDL)-treated macrophage cell line RAW 264.7 cells [31] and upregulates the transcriptional activity of PPAR*γ* [32].

In the past, studying SHP protein structure has been difficult due to the inherent insolubility of the SHP protein during its purification process. Recently, by using a maltose binding protein (MBP) fusion strategy, Zhi et al. generated a series of mouse MBP-SHP proteins that contain SHP N-terminal truncations and found that when SHP’s putative helices H1 and H2 were removed, MBP-SHP fusion proteins became highly soluble, making crystallization possible [33]. This study showed the crystal structure of SHP in a complex with the corepressor EID1. This reveals a conserved EID1-binding site at the N-terminus of SHP protein, where EID1 mimics helix H1 and becomes an integral part for the SHP protein LBD-fold [33]. As would be expected, the disruption of EID1-binding site affects SHP repressive function [33]. The identification of the SHP-EID1 complex and integral structural motifs is important as it provides a mechanism for SHP transcriptional repression and reveals a protein interface that regulates SHP repressive function.

## 4. Posttranslational Modifications of SHP Protein by Bile Acids

The wide variety of SHP-interacting partners indicates its versatility as a regulatory element in many cellular and physiological pathways. As a unique transcriptional repressor without known endogenous ligands, the identification of signaling pathways and transcriptional factors that affect SHP expression is essential for understanding the regulatory function of SHP in metabolism and disease and for future development of novel SHP-modulating therapeutic agents. Some major stress signaling pathways activate SHP including the mitogen-activated protein kinase/extracellular signal-regulated kinase 1/2 (MAPK/Erk1/2) pathway and the AMP-activated protein kinase (AMPK) pathway, while the phosphatidylinositol 3 kinase (PI3K) pathway represses SHP expression [34–37]. For example, one recent study shows that the inhibition of MEK1/2 pathway or the activation of PI3K pathway leads to SHP repression in metabolic conditions such as advanced nonalcoholic fatty liver disease (NAFLD) [38].

While SHP expression can be induced by numerous NRs and TFs such as steroidogenic factor-1 (SF-1), LRH-1, FXR, c-jun, HNF4*α*, ERR*γ*, E2A gene products (E47, E12, and E2/5), LXR*α*, ER*α*, sterol regulatory element-binding protein 1c (SREBP-1c), adaptor protein (AP1), PXR, PPAR*γ*, upstream stimulatory factor-1 (USF-1), and core circadian component CLOCK-BMAL1, no repressor for SHP expression has been identified until recently. FOXA1 and C/EBP*α* are reported as new repressors for SHP gene expression in advanced NAFLD [38].

Bile acids bind to FXR and induce SHP gene transcription; this is a well-known mechanism involved in bile acid homeostasis [2, 3]. Interestingly, recent studies show that bile acids, or bile acid-induced intestinal fibroblast growth factor 15 (Fgf15, FGF19 in human) signaling, induce post-translational modifications (PTMs) of SHP protein. These PTMs enhance SHP protein stability, nuclear localization, and protein interaction with corepressors, which profoundly modulate SHP regulatory function in diverse metabolic processes. For example, Miao et al. shows that SHP protein is rapidly degraded via the ubiquitin-proteasome pathway and that bile acids or FGF19 signaling increases hepatic SHP protein stability by inhibiting proteasome degradation in an ERK-dependent manner [22]. In addition, SHP is phosphorylated at Ser26 upon bile acid treatment, and mutation of this site dramatically decreases SHP stability [39]. Another study shows that bile acid treatment induces SHP protein methylation at Arg-57 by protein arginine N-methyltransferase 5 (PRMT5), which enhances SHP repressive activity by selectively increasing SHP protein interaction with repressive chromatin modifiers such as Brm, mSin3A, and histone deacetylase 1 (HDAC1) [40]. Relevant to this study, bile acids or FGF19 signaling activates an atypical protein kinase C*ζ* (PKC*ζ*) that phosphorylates SHP protein at Thr-55, which is important for the transcriptional repressor function of SHP in the regulation of metabolic target genes [41].

Extensive research in the last 2 decades has unveiled bile acids as key signaling molecules that control integrative metabolism and energy expenditure. A number of bile acid-activated signaling pathways have become attractive therapeutic targets for metabolic disorders [42]. As an orphan NR without known endogenous ligands, targeting PTMs of SHP by bile acids or FGF19 signaling to increase SHP repressive activity could be an effective therapeutic strategy for treating metabolic diseases.

## 5. Antitumor Role of SHP in Liver Cancer

HCC is one of the most deadly human cancers worldwide with few therapeutic options and poor prognosis. Improving our understanding of molecular mechanisms leading to HCC would provide new insights into developing novel treatments or prevention strategies. While it is well-known that chronic viral hepatitis-associated liver cirrhosis, nonalcoholic steato-hepatitis (NASH), ethanol consumption, hereditary diseases (*α*1 antitrypsin deficiency, hemochromatosis), and exposure to hepatotoxins (aflatoxin) are the major risk factors for HCC development [43], the inherent heterogeneity in HCC and its distinct subtypes that are linked to different potential oncogenic pathways still represent the major barriers for HCC study [44–46].

Hepatocarcinogenesis is a multistep process that involves the progressive accumulation of different genetic and epigenetic alterations leading to defective apoptosis and increased cell proliferation. Genetic alterations include oncogene activation and tumor suppressor loss. Epigenetic alterations include DNA methylation, histone modification, and deregulation of mirRNA [47]. Because NRs have been implicated in regulating the biology of a wide variety of cancers such as breast cancer, lung cancer, prostate cancer, and liver cancer, they are emerging targets for molecular diagnostic tests and cancer therapeutics [48]. Recently, it is becoming clear that SHP plays an antitumor role in the development of liver cancer. In this section we will summarize recent findings regarding the epigenetic silencing of SHP in human HCC and discuss the pleiotropic role of SHP in regulating cell proliferation, apoptosis, DNA methylation, and inflammation that are related to an antitumor role of SHP in the development of liver cancer.

### 5.1. Epigenetic silencing of SHP in human HCC

Aberrant gene promoter hypermethylation is an important mechanism leading to tumor suppressor gene silencing in HCC. Our earlier study showed that SHP expression is significantly diminished in human HCC pathologic specimens due to the epigenetic silencing caused by hypermethylation of cytosine-guanine dinucleotides (CpG) islands on SHP gene promoter [49]. Additionally, the methylation of the SHP gene promoter blocks nuclear receptor LRH-1-induced SHP gene transcription, which is reversed by demethylation of the SHP promoter [49]. Consistent with our study, a significantly lower level of SHP was shown in HCC when compared to nonmalignant liver tissue [50]. This study also reveals that the loss of SHP is more pronounced in fibrolamellar carcinoma than in typical HCC [50].

An association between SHP expression and HCC patient survival was recently established and revealed SHP as a positive prognostic factor. Conversely, an HCC patient with low SHP expression combined with a high expression of CDK4, MCM5, EXOCS1, CCNB1, BUB3, or BCL2L2 indicates a poor prognosis with a low chance of survival [51]. While bile acids or bile acids-mediated FGF19 signaling induces PTMs of SHP and increases SHP protein stability [39], whether these approaches could rescue SHP expression level in HCC and subsequently be used as effective therapeutic strategies for treating HCC still remains to be explored.

### 5.2. SHP in repression of cell proliferation in HCC

Uncontrolled proliferation is one of the fundamental hallmarks of cancer cells. Deeper investigations of SHP function may elucidate an important pathway through which tumor progression can be controlled. Our earlier study reveals a unique role of SHP in inhibiting cell growth through repressing Cyclin D1 expression, thereby providing a molecular basis for tumor suppressor function of SHP during HCC development [52]. *Shp*^−/−^ mice develop spontaneous HCCs at 12 to 15 months of age, which is strongly associated with enhanced cell proliferation and increased expression of Cyclin D1 [52]. Further support for SHP as a negative regulator of Cyclin D1 gene transcription is through its direct inhibition of nuclear receptor LRH1 recruitment to Cyclin D1 gene promoter. In agreement with SHP negatively regulating cellular growth, the immortalized *Shp*^−/−^ fibroblasts form tumors in nude mice [52].

Yes-associated protein (YAP) is an effector of the Hippo kinase tumor-suppressor pathway which plays a critical role in promoting cholangiocyte and hepatocyte proliferation and survival during embryonic liver development and hepatocellular carcinogenesis [24]. *Fxr*^−/−^*Shp*^−/−^ double knockout mice exhibit a severe defect in bile acid homeostasis with the increase of progenitor cell proliferation, YAP activation, and spontaneous liver cancer formation [44]. In addition, the phenotype in *Fxr*^−/−^*Shp*^−/−^ double knockout mice mirrors mice with loss of Hippo kinases or overexpression of YAP. Therefore, YAP activation triggered by elevated bile acids level via the loss of SHP promotes spontaneous HCC formation in SHP-deficient liver.

### 5.3. SHP in regulation of apoptosis in HCC

Apoptosis is important to maintain tissue homeostasis. As previously mentioned, cancer cells elude this pathway and continuously proliferate; thus, it has been hypothesized and proven that, in dysplasia and the pathogenesis of HCC, regulation of apoptosis is altered [53]. SHP is a critical component of apoptotic signaling as evidenced by the decrease of apoptosis in *Shp*^−/−^ mouse livers and the hypersensitization of hepatocyte-specific SHP-overexpression mice to Fas-mediated apoptosis [54].

The synthetic retinoid-like compounds 6-[3-(1-adamantyl)-4-hydroxyphenyl]-2-naphthalenecarboxylic acid (CD437/AHPN) and 4-[3-(1-adamantyl)-4-hydroxyphenyl]-3-chlorocinnamic acid (3-Cl-AHPC) are effective apoptosis inducers in malignant cells. Accumulating evidence indicates that SHP is an essential mediator for the proapoptotic activities of AHPN and 3-Cl-AHPC. For instance, in human leukemia cell lines HL-60R, KG-1, and breast carcinoma cell line MDA-MB-468, AHPN or 3-Cl-AHPC binds to SHP protein and promotes the formation of a corepressor complex containing Sin3A, NR corepressor (N-CoR), histone deacetylase 4, and heat shock protein 90 (HSP90) [55]. SHP deficiency compromises the proapoptotic activities of AHPN or 3-Cl-AHPC [55], indicating that SHP is required for AHPN and 3-Cl-AHPC to exert their proapoptotic activities. In addition, AHPN promotes SHP protein targeting to mitochondria while interacting with antiapoptotic protein BCL2, leading to the disruption of the BCL2/BID complex and the release of proapoptotic protein BID. As a consequence, the free BID protein then targets mitochondria and leads to the activation of apoptosis [54]. The above findings provide the intriguing possibilities to apply AHPN or 3-Cl-AHPC to manipulate SHP expression or localization for cancer treatments.

In contrast, an antiapoptotic role of SHP has been shown in Nur77-mediated apoptosis. Nur77 induces apoptosis in multiple cell types including hepatocytes. In anti-Fas antibody (CH11)-mediated hepatocyte apoptosis, SHP exerts an antiapoptotic role through interaction with the transcriptional coactivator CBP and releases CBP from Nur77 promoter, leading to a transcriptional repression of Nur77 and, ultimately, a reduction in apoptosis [56]. Consistent with this finding, overexpression of SHP decreases, whereas knockdown of SHP increases the transcription of Nur77 in human liver cancer HepG2 cells [56]. In addition, SHP is significantly increased in the interferon *γ* (IFN*γ*)/CH11-resistant HepG2 cells, whereas no SHP is detected in the IFN*γ*/CH11-sensitive liver cancer SNU354 cells [56]. Alternatively, constitutive expression of SHP in SNU354 cells results in a resistance to IFN*γ*/CH11-induced apoptosis [56].

The discrepancy of SHP’s role in cell apoptosis could be due to the differences in cell types and cell treatment conditions. For example, our unpublished work suggests that SHP protects hepatocytes from nutrient starvation-induced apoptosis but enhances free fatty acid-associated lipotoxicity-induced apoptosis. Further study of the detailed mechanism is currently undergoing.

### 5.4. SHP in the regulation of DNA methylation in HCC

Tumor suppressor gene silencing by promoter hypermethylation is mainly controlled by three DNA methyltransferases (Dnmt1, Dnmt3a, and Dnmt3b). Dnmt 1 plays a critical role in maintaining CpG methylation. While upregulation of Dnmt 1 and its associated aberrant gene silencing of tumor suppressors are frequently observed in human HCC [57, 58], the mechanism of Dnmt1 upregulation remains elusive. Recent studies reveal SHP as a potent repressor of Dnmt1 transcription through two mechanisms. On one hand, nuclear receptor ERR*γ* acts as a transcriptional activator of Dnmt1 by directly binding to its response elements (ERE1/ERE2) on the Dnmt1 promoter. Here, SHP interacts with ERR*γ* and diminishes ERR*γ* recruitment to the Dnmt1 promoter, ultimately altering the conformation of local chromatin from an active mode to an inactive mode that inhibits Dnmt1 transcription [59]. On the other hand, SHP inhibits zinc-dependent induction of Dnmt1 by antagonizing metal-responsive transcription factor-1 (MTF-1) [60]. Zinc treatment induces Dnmt1 transcription by increasing the occupancy of MTF-1 on the Dnmt1 promoter while decreasing SHP expression. SHP, in turn, represses MTF-1 expression and abolishes zinc-mediated changes in the chromatin configuration of the Dnmt1 promoter, ultimately resulting in the transcriptional repression of Dnmt1. In this study, an inverse correlation between increased DNMT1 expression and decreased SHP expression in human HCC is also established. All of these studies indicate that loss of SHP could lead to the upregulation of DNMT1 resulting in aberrant tumor suppressor genes silencing in HCC, which contributes to HCC development under SHP-deficient conditions.

### 5.5. SHP in inflammation in HCC

Persistent inflammation in the absence of infection increases the risk and accelerates the development of cancer [61]. Previous work has demonstrated that NRs actively inhibit inflammation through repressive interactions with various transcriptional factors such as NF-*κ*B [62]. Recently, an inhibitory role of SHP in inflammation was appreciated. Li et al. shows that upregulation of SHP by FXR ligand GW4064 inhibits interleukin IL-1*β*-induced inducible nitric oxide synthase (iNOS) and cyclooxygenase-2 (COX-2) production in vascular smooth muscle cells [63], which is the first study to provide a link between SHP and inflammation.

NF-*κ*B/Rel proteins including NF-*κ*B2 p52/p100, NF-*κ*B1 p50/p105, c-Rel, RelA/p65, and RelB are dimeric transcription factors that control genes in innate and adaptive immunity and inflammation [64]. In 2011, a relationship between SHP and NF-*κ*B inflammatory pathway was suggested [5]. Here, SHP inhibits Toll-like receptor (TLR)-induced inflammatory responses in innate immune cells through a biphasic interaction with two cytoplasmic partners of TLR4 pathway, TRAF6 and NF-*κ*B subunit p65 [5].

In addition, a novel role for fenofibrate in inhibiting systemic inflammatory responses in a SHP-dependent fashion was recently identified [65]. In this study, fenofibrate induces SHP expression and increases survival in an animal model of sepsis by increasing the expression of mitochondrial uncoupling protein 2 (UCP2). UCP2 is required to suppress inflammatory responses in this model through the modulation of mitochondrial reactive oxygen species (ROS) production [65].

The inflammasome, a large multimeric protein complex, is important for the activation of caspase-1 and maturation of the proinflammatory cytokines interleukin (IL)-1*β* and IL-18. While inflammasome activation plays a key role in host defense against a variety of pathogens, its excessive and uncontrolled activation may be damaging to the host, resulting in autoimmune diseases. SHP “fine-tunes” the activation of the pyrin domain-containing 3 (NLRP3) inflammasome by binding directly to NLRP3 and subsequently prevents excessive inflammatory responses [66].

HCC develops on a background of chronic inflammation mainly triggered by exposure to infectious agents (hepatitis virus) or to toxic compounds (alcohol) [61]. While the molecular links between inflammation and HCC are not completely elucidated, it is becoming clear that inflammatory cells in the tumor microenvironment are indispensable participants in the neoplastic process, fostering proliferation, survival, and migration [67]. SHP plays a fundamental role as a negative regulator of inflammation. Therefore, SHP loss would lead to an increase in chemokine and cytokine production that could promote the development of liver cancer. As direct evidence, *Shp*^−/−^ mice have increased levels of chemokines and cytokines in serum together with the activation of signal transducer and activator of transcription 3 and 5 (STAT3 and STAT5) inflammatory signaling pathways in the liver (our unpublished data) and develop spontaneous hepatocellular carcinomas at 12 to 15 months of age [52].

### 5.6. Loss of SHP results in liver cancer development

**Figure 3:**
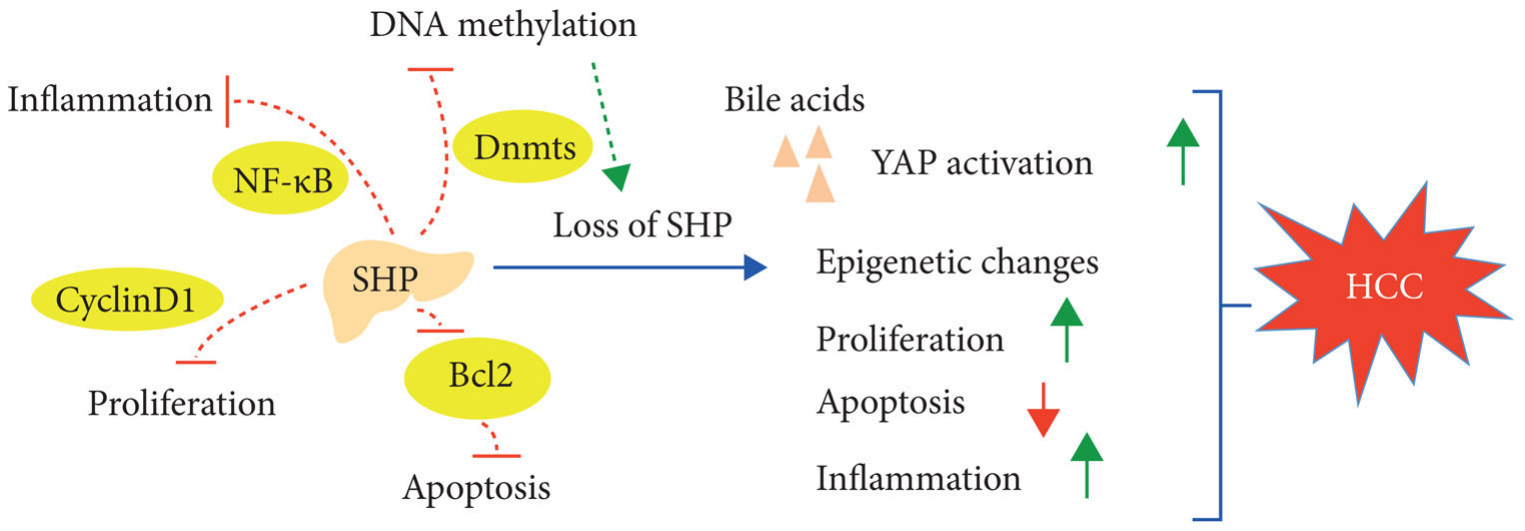
SHP loss results in HCC development. SHP is silenced in HCC due to promoter hypermethylation. Reduced SHP leads to upregulation of Dnmts predicted to further enhance the methylation and silencing of SHP and other tumor suppressor genes. This would generate a self-amplifying cycle of epigenetic events leading to long-term transcriptional repression of SHP and other tumor suppressors. In turn, this leads to an increase in cell proliferation through Cyclin D1 activation and defective apoptosis through the loss of inhibition on antiapoptotic protein Bcl2. Additionally, SHP loss increases bile acid levels, resulting in YAP activation. Loss of SHP also induces the activation of inflammatory genes and signaling pathways. Collectively, these events coordinately promote the development of HCC. Activation is shown as a green arrow and inhibition is shown as a red line.

As we have discussed above, the SHP gene is hypermethylated and silenced in HCC. A decrease in SHP leads to upregulation of Dnmts that is predicted to further enhance the methylation and silencing of SHP and other tumor suppressor genes. This would generate a self-amplifying cycle of epigenetic events leading to the long-term transcriptional repression of SHP and other tumor suppressors, which leads to increase of cell proliferation through the activation of Cyclin D1 and defective apoptosis through the loss of inhibition on antiapoptotic protein BCL2. Additionally, the loss of SHP increases bile acid levels, resulting in YAP activation. Loss of SHP also induces the activation of inflammatory signaling pathways. Collectively, all of these events coordinately promote the development of HCC. The schematic of loss of SHP results in liver cancer is summarized in Figure 3.

## 6. Drug Targets for SHP

FXR ligands bile acids and GW4064, RXRs/RARs ligands such as all-retinoic acids, 9-cis-retinoic acids, are known to induce SHP expression [2, 3, 68, 69]. Synthetic retinoid-like compounds, AHPN and 3-Cl-AHPC, induce SHP expression through the increase of LRH1 recruitment to the SHP gene promoter [54]. SHP protein contains a conserved ligand-binding domain, suggesting a possibility that its activity could be regulated by natural or synthetic ligands. Indeed, AHPN and 3-Cl-AHPC bind to SHP protein and promote SHP interaction with LXXLL-containing peptides [55, 70]. These findings suggest the intriguing possibilities of manipulating SHP for the treatment of liver cancer and several metabolic diseases such as hypercholesterolemia, obesity, diabetes, and fatty liver disease.

## 7. Conclusion and Perspective

SHP is a unique NR distinct from other conventional NRs in both structure and function. The fact that SHP acts as a critical transcriptional corepressor in diverse metabolic processes provides potential means to develop SHP-targeted therapeutics for metabolic diseases. SHP acts as a tumor suppressor in the development of liver cancer, and the loss of SHP results in genome wide aberrant DNA methylation, indicating that manipulation of SHP function may have implications for cancer treatment. Of particular interest, a number of retinoid-like compounds bind to SHP and enhance SHP repressive function in liver cells. This raises the possibility of developing synthetic agonists that selectively activate SHP as therapeutics for liver cancer and several metabolic diseases. Major challenges for future research are to determine whether endogenous SHP ligands exist and to develop more efficacious and specific synthetic SHP ligands for pharmaceutical applications.
